# Minute Hæmorrhages in the Pia Mater

**Published:** 1883-12

**Authors:** T. Steele Sheldon

**Affiliations:** Assistant Medical Superintendent, Somerset and Bath Asylum


					MINUTE HEMORRHAGES IN THE PIA MATER.
By T. Steele Sheldon, M.B. Lond., Assistant
Medical Superintendent, Somerset and Bath Asylum.
Amelia C., aged 29, was admitted into the Somerset and
Bath Asylum in January, 1881. Two years previously
p 2
226
HEMORRHAGES IN THE PIA MATER.
she is said to have had a " severe attack of intermittent
ague and fever, and has had pain in the head ever since."
She was suffering from melancholia, was very suicidal,
and frequently refused food. In December, 1881, she
contracted enteric fever, and intercurrent pneumonia
proved fatal on January 26th, 1883. During the illness
she was for the most part acutely conscious, rational and
coherent. On January 25th the temperature fell suddenly
to 970; in the evening it rose to 102°. On the 26th she
was very restless, constantly made sweeping movements
of the arms above her head, cried out at intervals, and
was delirious. She died in the evening.
A utopsy.—The brain weighed 475- oz. The Pacchionian
bodies were largely developed. The convolutions were
intricate but somewhat atrophied, the sulci being wide
and marked out under the arachnoid by excess of fluid.
There was a remarkable difference in the surface appearance
of the hemispheres; on the left side the piaarachnoid
was thin, transparent, and marked only by
injected meandering vessels; on the right side the membrane
over the superior and middle frontal convolutions,
the upper half of the ascending frontal and parietal gyri,
and part of the lower parietal lobule, was opaque, soft,
reddish, and thickened. On removing it the grey matter
below was soft, highly congested, and on the slightest
provocation came away with the pia mater. The affected
membrane was, moreover, marked by numerous conspicuous
bright-red dots, about the size of the head of a
small pin, or less. Microscopic examination proved them
to be spherical masses of red blood-corpuscles, abutting,
like bosses, against the wall of the vessels; they were
frequently arranged in pairs, the extravasation having
occurred at opposite points in the same section of a
GOUTY INFLAMMATION OF THE TESTIS.
227
vessel. There was no other noteworthy appearance in
the brain.
Remarks.—The above - described morbid appearances
would seem to be rare, judging from the little I can find
bearing on the subject in the pathological works I have at
my disposal. Cornil and Ranvier give a description of
the pia mater in cerebral rheumatism and other forms of
cerebral congestion accompanied by delirium, among
them, typhoid fever.* This description, corresponding
in the main with the account given above, is the only
reference I have been able to find. It is curious that the
lesion was only on one side of the brain; on the affected
side it was also limited, that area of the pia-arachnoid
being involved which is so constantly the seat of chronic
thickening and opacity; speaking roughly, the motor area
and the frontal lobe.
* Pathological Histology, p. 578 (Eng. Trans.)

				

